# Processing of *Chlamydia abortus* Polymorphic Membrane Protein 18D during the Chlamydial Developmental Cycle

**DOI:** 10.1371/journal.pone.0049190

**Published:** 2012-11-08

**Authors:** Nick M. Wheelhouse, Michelle Sait, Kevin Aitchison, Morag Livingstone, Frank Wright, Kevin McLean, Neil F. Inglis, David G. E. Smith, David Longbottom

**Affiliations:** 1 Moredun Research Institute, Edinburgh, Midlothian, United Kingdom; 2 Biomathematics and Statistics Scotland, Dundee, United Kingdom; 3 Moredun Proteomics Facility, Moredun Research Institute, Edinburgh, Midlothian, United Kingdom; 4 Institute of Infection, Immunity and Inflammation, College of Medical, Veterinary and Life Sciences, University of Glasgow, Glasgow, United Kingdom; University of California Merced, United States of America

## Abstract

**Background:**

*Chlamydia* possess a unique family of autotransporter proteins known as the Polymorphic membrane proteins (Pmps). While the total number of *pmp* genes varies between *Chlamydia* species, all encode a single *pmpD* gene. In both *Chlamydia trachomatis* (*C. trachomatis*) and *C. pneumoniae*, the PmpD protein is proteolytically cleaved on the cell surface. The current study was carried out to determine the cleavage patterns of the PmpD protein in the animal pathogen *C. abortus* (termed Pmp18D).

**Methodology/Principal Findings:**

Using antibodies directed against different regions of Pmp18D, proteomic techniques revealed that the mature protein was cleaved on the cell surface, resulting in a100 kDa N-terminal product and a 60 kDa carboxy-terminal protein. The N-terminal protein was further processed into 84, 76 and 73 kDa products. Clustering analysis resolved PmpD proteins into three distinct clades with *C. abortus* Pmp18D, being most similar to those originating from *C. psittaci*, *C. felis* and *C. caviae*.

**Conclusions/Significance:**

This study indicates that *C. abortus* Pmp18D is proteolytically processed at the cell surface similar to the proteins of *C. trachomatis* and *C. pneumoniae*. However, patterns of cleavage are species-specific, with low sequence conservation of PmpD across the genus. The absence of conserved domains indicates that the function of the PmpD molecule in chlamydia remains to be elucidated.

## Introduction

The family *Chlamydiaceae* are a diverse group of obligate intracellular Gram-negative bacteria that cause a range of pathogenic conditions in a wide variety of host species [1]. All known members share a similar and distinct biphasic developmental cycle, which is initiated with the entry of the infectious form of the organism, the elementary body (EB), into the host cell where it resides within a vacuole known as an inclusion. The EB undergoes conversion to the metabolically active reticulate body (RB), which replicates through binary fission. Towards the end of the cycle (48 to 72 hours following infection) the RBs re-condense to EBs before both the inclusion and host cell are lysed, allowing the release of the infective organisms to infect neighbouring cells [1].

The Type V or autotransporter (AT) secretion system comprises the largest family of proteins found across pathogenic Gram-negative bacteria. Classical AT structure is characterised by the presence of three separate functional domains; a cleavable N-terminal signal sequence; a passenger (effector) domain and a carboxy-terminal β-barrel translocator domain [2]. While the overall structure and organisation of ATs is similar across bacterial species, the function of the effectors vary. However, many of these proteins have been identified as virulence factors involved in bacterial pathogenesis. Chlamydia possess a unique family of proteins that have been identified as ATs (known as the Polymorphic membrane proteins (Pmps)) [3]. Pmps were first identified in *Chlamydia abortus* (*C. abortus)* due to their immuno-reactivity with convalescent sheep sera [4], [5], and have now been identified in all of the pathogenic *Chlamydia* spp. Significant heterogeneity of Pmp gene carriage has been observed between chlamydial species. Genome sequencing of *C. trachomatis* has revealed the presence of 9 *pmp* genes (termed A-I) [6] while 21, 16, 18, 17, 21 and 20 *pmps* have been identified in *C. pneumoniae*, *C. pecorum*, *C. abortus*, *C. caviae, C. psittaci* and *C. felis* respectively [7]–[11].

The PmpD proteins of both *C. trachomatis* and *C. pneumoniae* are expressed throughout the chlamydial developmental cycle. Pmps are highly immunogenic and there has been much interest in their exploitation as vaccine and diagnostic candidates. Recently, attention has been focussed upon PmpD due to the ability of antibodies raised against it to neutralize the infectivity of both *C. trachomatis* and *C. pneumoniae in vitro*, and the level of seroconversion to the protein observed in *C. trachomatis* infected individuals. PmpD is cleaved, and it has been hypothesised that this may permit secretion of specific effector peptides into host cells or within the inclusion [12]. However, little is understood about the function or processing of the PmpD molecule in any other *Chlamydia* spp.

This study focuses on *C. abortus*, the causative agent of ovine enzootic abortion (OEA), a disease of significant economic importance to the sheep rearing industry and of concern to human health, particularly pregnant women [13]. The *pmp* genes are grouped within specific families by their phylogenetic similarity with the originally identified *C. trachomatis pmps* [14]. However, due to the expansion in *pmp* gene number in *C. abortus*, the *pmps* have additionally been numbered sequentially by their position in genome, with the *C. abortus* PmpD protein being termed Pmp18D [8]. Given the potential conservation of roles of the PmpD molecules across chlamydial species studied to date and their potential as diagnostic or vaccine candidates, this study was carried out to investigate the structural features and processing of the *C. abortus* Pmp18D molecule.

## Materials and Methods

### *C. abortus* propagation

McCoy cells were obtained from the European Collection of Cell Cultures (ECACC, Salisbury, UK) and maintained in RPMI1640 medium supplemented with 10% heat inactivated fetal calf serum (PAA Laboratories Ltd, Yeovil, Somerset, UK). The *C. abortus* strain S26/3 was propagated in McCoy cells, according to a previously published protocol [15].

### Antibodies and Western blotting

Rabbit polyclonal antibodies were generated against *C. abortus* S26/3 Pmp18D peptides: N-Pmp18D (N terminal region of Pmp18D) EKPIHAQGPKKGETD (amino acids (aa) 67–81); Mid-Pmp18D (middle domain region of Pmp18D) DPNAKPTEKIESPTS (aa 1052–1066) (both Eurogentec, Southampton, UK); C-Pmp18D (carboxy terminus region of Pmp18D) CQPNLGGSKGSWDSR (aa 1357–1370) (Genscript USA Inc., Piscataway, NJ, USA). In addition, the mouse anti-Omp-1 mAb 4/11 [16] was used for the detection of *C.abortus* Omp-1. Total cell lysates were prepared by scraping infected S26/3 infected McCoy monolayers and pelleting at 12,000 rpm in a microcentrifuge at 24, 48 or 72 h post-infection (p.i.). The resulting pellets were resuspended in 1 ml 1× Laemmli loading buffer [17]. After brief sonication and boiling for 5 mins, proteins were separated on 4–12% NuPAGE gels (Life Technologies, Paisley, UK), transferred onto nitrocellulose and Western blotted. Detection was accomplished using the ECL-advance system and results were visualized using the LAS400 Quantitative imaging system (both GE Healthcare, Chalfont St Giles, Buckinghamshire, UK). Molecular masses of recognized protein products were calculated using a standard curve, calculated from the Rf values obtained for SeeBlue® Plus2 markers (Life Technologies) and using ImageQuant TL 1D-PAGE analysis software (GE Healthcare).

### Pmp18D solubility in infected McCoy cells

To investigate if Pmp18D protein fragments are released or retained on the surface of the *C. abortus* outer membrane, soluble and insoluble protein fractions from *C. abortus* infected cells were prepared essentially as previously described [12]. Briefly, infected 225 cm^2^ flasks of McCoy cell monolayers were harvested at 48 h post-infection and washed with 5 ml hypotonic swelling buffer (15 mM KCl, 1.5 mM Mg(Ac)_2_ and 10 mM HEPES, pH 7.4) containing protease inhibitors (Sigma Aldrich, Dorset, UK; P9549). Cells were gently lysed by 20 strokes using a Dounce homogenizer. The homogenate was briefly centrifuged for 5 mins at 200× *g* and the resulting supernatant further centrifuged at 125,000× *g* (SW55Ti rotor Beckman Coulter). The supernatant (soluble material) was removed and the protein precipitated with 3 volumes of acetone at −20°C. The pellet (insoluble material) was washed twice with ice-cold PBS containing protease inhibitors before lysis in 1 ml 1× Laemmli loading buffer. The precipitated supernatant material was subsequently lysed in the same volume of 1× Laemmli buffer. Samples were analysed by Western blotting, as described above.

### Identification of major high molecular weight cleavage products

Identification of the major passenger cleavage products was accomplished using 2 independent proteomic methods:

### Liquid chromatography-electrospray ionisation-tandem mass spectrometry (LC-ESI-MS/MS)

For the initial identification of the cleavage products, replicate 100 µl samples of S26/3 infected total cell lysate were separated on a 7% 16 cm Slab gel (Hoefer SE600, GE Healthcare). The gel was cut into two equal pieces. One half was blotted onto nitrocellulose using a semi-dry blotting apparatus prior to Western blotting using the 3 antibodies directed against the different regions of Pmp18D. The second half of the gel was stained with Simplyblue™ SafeStain (Life Technologies). The position of the identified bands was determined and the gel slices excised from the gel. Proteins were destained and reductively alkylated using DTT and iodoacetamide. Gel slices were then digested overnight with trypsin (Porcine trypsin, Promega, Hants, UK) at 37°C. Samples were transferred to low-protein-binding HPLC sample vials and stored at 4°C until required for LC-ESI-MS/MS analysis. Liquid chromatography was performed using an Ultimate 3000 nano-UHPLC system (Dionex) comprising a WPS-3000 well-plate micro auto sampler, a FLM-3000 flow manager and column compartment, a UVD-3000 UV detector, an LPG-3600 dual-gradient micropump and an SRD-3600 solvent rack controlled by Chromeleon chromatography software (Dionex). A micro-pump flow rate of 246 µl/min was used in combination with a cap-flow splitter cartridge, affording a 1/82 flow split and a final flow rate of 3 µl/min through a 5 cm×200 µm ID monolithic reversed phase column (Dionex) maintained at 50°C. Samples of 4 µl were applied to the column by direct injection. Peptides were eluted by the application of a 15 min linear gradient from 8–45% solvent B (80% acetonitrile, 0.1% (v/v) formic acid) and directed through a 3 nl UV detector flow cell. LC was interfaced directly with a 3-D high capacity ion trap tandem mass spectrometer (amaZon ETD, Bruker Daltonics) via a low-volume (50 µl/min maximum) stainless steel nebuliser (Agilent, cat. no.G1946-20260) and ESI. Parameters for tandem MS analysis were set as previously described [18] with minor modifications as detailed below.

Database Mining: Deconvoluted MS/MS data in Mascot generic file (mgf) format was imported into ProteinScape™ analysis software (Bruker Daltonics) and searched against the *C.abortus*_NCBInr database utilising the Mascot (Matrix Science) search algorithm. The data was also searched specifically against the cognate pmp18D protein sequence using the Sequence-Editor function of Biotools™ analysis software (Bruker Daltonics). The interpretation of MS/MS data was performed in accordance with published guidelines [19]. To this end, fixed (carbamidomethyl C) and variable (deamidation N,Q and oxidation M) modifications were selected, and mass tolerance values for both MS and MS/MS were set at ±0.5 Da allowing for one 13C isotope and a single missed cleavage. Molecular weight search (MOWSE) scores and percentage coverage values attained for individual protein identifications were inspected manually and considered significant only if a) two peptides were matched for each protein, and b) each peptide contained an unbroken “b” or “y” ion series of a minimum of four amino acid residues.

### Matrix-assisted laser desorption/ionization- time of flight mass spectrometry (MALDI-ToF MS)

Infected McCoy cell monolayers were disrupted with sterile glass beads, and was briefly sonicated, prior to centrifugation at 1000 rpm for 10 minutes in a JA-14 rotor (Beckman Coulter), to remove gross cellular debris. The supernatant was removed and centrifuged at 12000 rpm for 40 minutes at 4°C using a JA-16.250 rotor. The pellet was resuspended in 5 ml 2% sarcosyl/PBS by brief sonication before incubation at 37°C for 30 minutes. The suspension was then centrifuged at 120,000× *g* using a SW55Ti rotor for 60 minutes at 4°C. The supernatant was removed and used for Pmp18D purification. Immunoprecipitation was carried out using Protein G Dynabeads® (Life Technologies) coupled to Pmp18D antibodies, as per the manufacturer's instructions. The final antibody:protein complex was disassociated by the addition of Laemmli loading buffer and heating at 95°C. Proteins were resolved on NuPAGE® polyacrylamide gels and visualised using Simplyblue™ SafeStain (both Life Technologies). Gel slices were treated as described for LC-ESI-MS-MS analysis. Digests were analysed on a Bruker Ultraflex II MALDI-ToF-ToF mass spectrometer (Bruker Daltonics), scanning the 600 to 5000 dalton region in reflectron mode producing monoisotopic resolution. The spectra generated were mass calibrated using known standards. Masses obtained were then database searched using the MASCOT search algorithm.

### Bioinformatic analysis of PmpD

PmpD amino acid sequences were obtained from GenBank and NCBI conserved domain searches were performed to define passenger-, M- and autotransporter-domains. PmpD sequences from the passenger-, and combined M- and autotransporter-domains were aligned using MUSCLE [20], and checked by mapping YASPIN secondary structure predictions [21] before being joined into a single alignment. Regions which could not be unambiguously aligned were eliminated using GBlocks v 0.91b [22], resulting in 1032 positions being analyzed (59% of the original 1726 positions). Bayesian dendrograms were generated using MrBayes software [23], launched from the TOPALi v2.5 package [24] using the CPRev+I+G substitution model that was determined to be the model of best fit, based on the BIC criterion. Trees were generated using Markov chain Monte Carlo (MCMC) settings of 2 runs of 625,000 generations with a burn-in of 125,000 generations with trees sampled every 100 runs.

## Results

### Expression of Pmp18D

**Figure 1:**
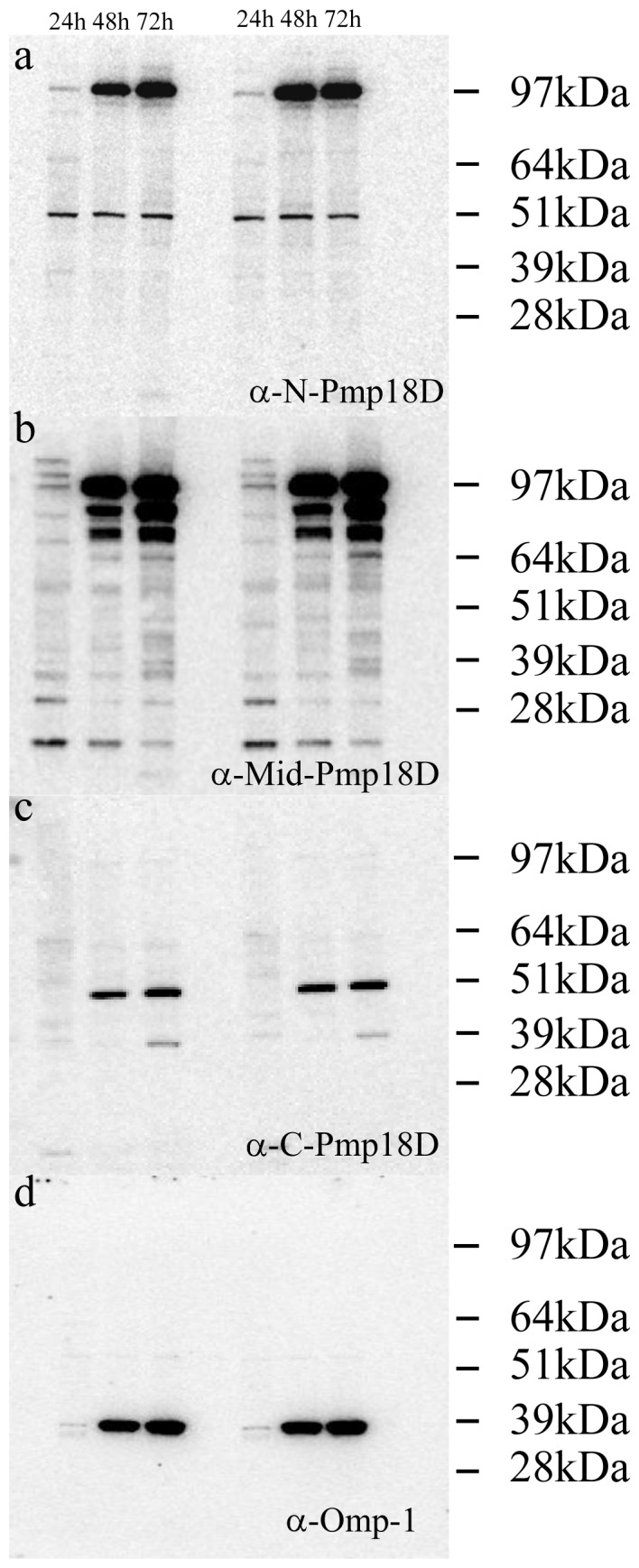
Western blot demonstrating an increase in Pmp18D expression and proteolytic cleavage over the 72 h *C. abortus* developmental cycle using: a) anti-N-Pmp18D, b) anti-Mid-Pmp18D, c) anti-C-Pmp18D pAbs and d) anti-Omp-1 mAb 4/11.

Western blot analysis using all 3 anti-Pmp18D antibodies demonstrated that expression of Pmp18D was time-dependent and correlated with an increase in Omp-1 levels over time (Figure 1). At any of the analyzed time points, using both the N-Pmp18D and Mid-Pmp18D antibodies, little if any intact 160 kDa molecular mass protein (which would correspond to the mature Pmp18D molecule) could be observed. The most highly immunoreactive protein among the identified bands was a molecule of approximately 94 kDa that was recognised by both N-Pmp18D and Mid-Pmp18D (Figures 1a and 1b respectively) and a 50 kDa protein recognised by the C-Pmp18D (Figure 1c) antibody alone. Additional protein bands at approximately 84 kDa and a doublet of a 76 kDa and 73 kDa were also routinely recognised by the Mid-Pmp18D antibody, particularly by 72 h p.i. (Figure 1b). However none of these additional bands were recognised by the N-Pmp18D antibody (Figure 1a).

### Pmp18D solubility in infected McCoy cells

**Figure 2:**
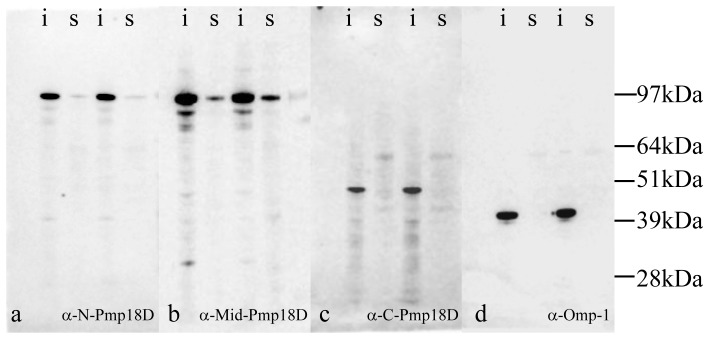
Western blot demonstrating the presence of the majority of Pmp18D in the insoluble material after sonication in PBS 48 h post-infection: a) anti-N-Pmp18D, b) anti-Mid-Pmp18D, c) anti-C-Pmp18D and d) anti-Omp-1 mAb 4/11.

To determine whether the Pmp18D passenger is secreted from *C. abortus* or retained on the bacterial surface, soluble and insoluble cell fractions were prepared from *C. abortus*-infected McCoy cells at 48 and 72 h p.i.. At both time points Omp-1 could be only identified in the insoluble fraction (Figure 2d), as could the 50 kDa band identified by the C-Pmp18D antibody (Figure 2c). The greater proportion of the protein bands recognised by both N-Pmp18D and Mid-Pmp18D antibodies was identified solely in the insoluble fraction. However, a small proportion of the 94 kDa product could also be detected in the soluble material (Figures 2a and 2b).

### Proteomic identification of Pmp18D products

Proteomic analysis was carried out to identify the protein bands recognised by the different antibodies targeting the various regions of Pmp18D. The passenger domain was found to be readily soluble in 2% sarkosyl (Figure S1) and the protein identification of these N-terminal bands was successfully accomplished using two independent techniques, LC-ESI-MS-MS and MALDI ToF MS using both N-Pmp18D and Mid-Pmp18D antibodies. Immunoprecipitation prior to MALDI ToF MS using the C-Pmp18D antibody was not successful and so only LC-ESI-MS-MS could be used to identify bands recognised using this antibody.

While 4 bands could be identified by Western blot using the Mid-Pmp18D antibody, only the protein sequence of the 2 most immunoreactive protein bands at approximately 94 kDa and 84 kDa could be determined by LC-ESI-MS-MS, and these were the only 2 proteins recovered by immunoprecipitation prior to MALDI-ToF MS analysis. These combined analyses gave broadly similar results, although peptide coverage with MALDI-ToF MS analysis appeared to be slightly greater. MALDI-ToF MS analysis identified the same peptide at the carboxy-terminus of both proteins, NAKPTEK^1060^ and this was confirmed by LC-ESI-MS-MS. The first detected N-terminal peptide sequence of the larger 94 kDa protein fragment, recognised by both N-Pmp18D and Mid-Pmp18D, was identified as ^67^EKPIHAQ by MALDI ToF MS and as ^76^KGETDQ by LC-ESI-MS-MS, giving a predicted molecular mass for this protein fragment of approximately 104 kDa based on sequence coverage (Figure S2a). Both methods identified the same initial peptide at the N-terminus of the predicted 84 kDa protein, with peptide coverage starting from ^215^LVDGCE, indicating that it is a product of the initial larger protein with cleavage at the N-terminus and the removal of approximately 150 amino acids (Figure S2b) giving an actual predicted molecular mass of 87.5 kDa.

**Figure 3:**
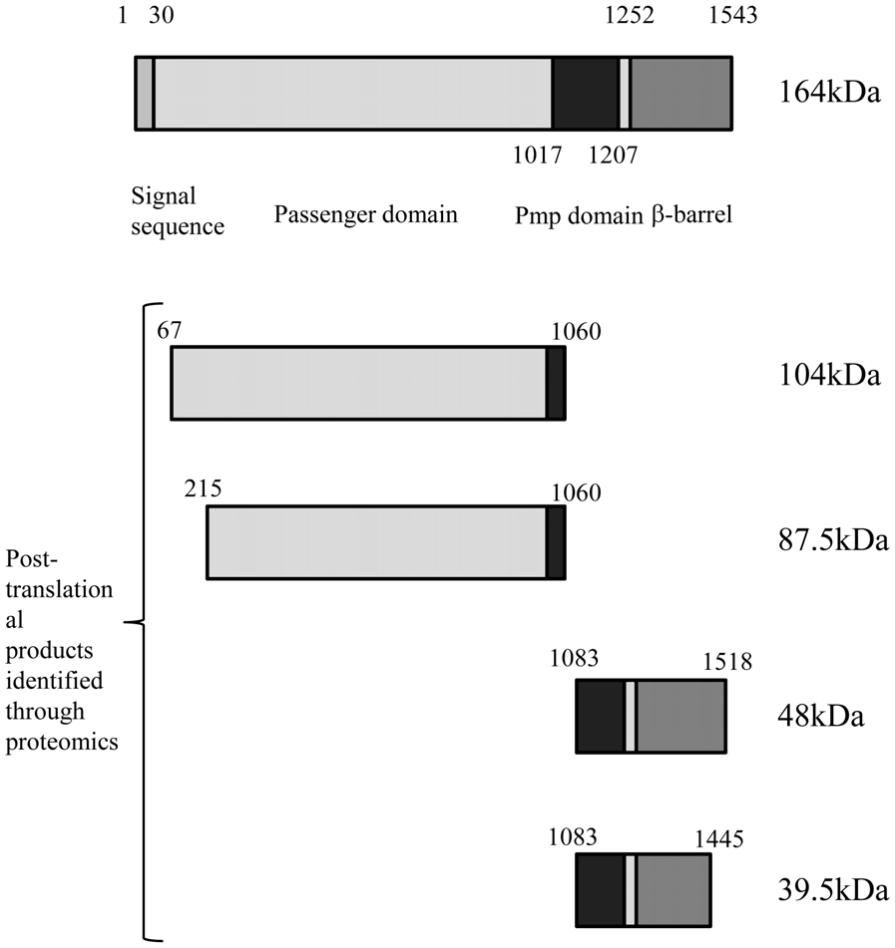
Schematic representation of Pmp18D cleavage products as revealed following proteomic analysis of protein fragments.

No protein could be recovered after immunoprecipitation using the carboxy antibody. However, LC-ESI-MS/MS analysis demonstrated sequence coverage of the 50 kDa fragment identified by the carboxy antibody from ^1083^TLADIN to LNCGMR^1518^ with a predicted molecular mass based on sequence coverage of 48 kDa (Figure S2c). A further cleavage product of the carboxy-terminal barrel was also identified with an initial peptide at ^1083^TLADIN with sequence coverage obtained to EHNYSR^1445^ (Figure S2d). The sizes of the predicted proteins remain approximate and are perhaps slightly underestimated as the peptides identified using both these techniques may not provide the actual first or last peptide sequences of the analysed proteins. A schematic diagram of the approximate cleavage sites for each of the identified PmpD protein fragments is shown in Figure 3.

### Bioinformatic analysis of PmpD

To determine whether the cleavage products observed in *C. abortus* Pmp18D and other *Chlamydia* spp occurred in conserved regions or motifs of the PmpD molecule, we performed an alignment and clustering analysis of PmpD protein sequences from the nine *Chlamydia* spp.

**Figure 4:**
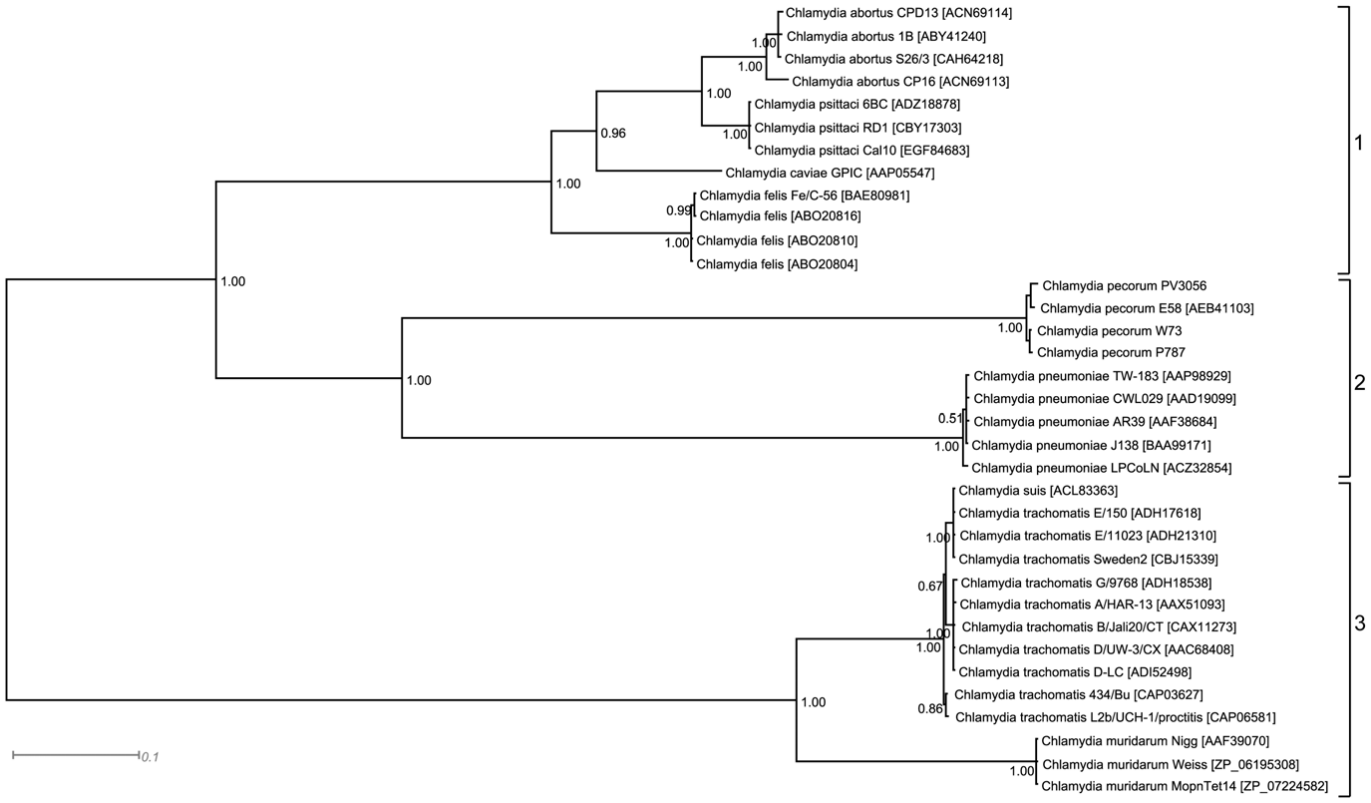
Bayesian dendrogram showing evolutionary relatedness of PmpD amino acid sequences originating from the nine *Chlamydia* species. Accession numbers of the amino acid sequences are indicated to the right of the strain name. The posterior probability of discrete groupings is indicated by the number at the node. The 3 PmpD clusters (cluster 1: *C. abortus*, *C. psittaci*, *C. caviae* and *C. felis*; cluster 2: *C. pneumoniae* and *C. pecorum*; and cluster 3: *C. trachomatis*, *C. suis* and *C. muridarum*) that were identified in this study are indicated to the right of the dendrogram.

Clustering analysis of PmpD identified in the nine *Chlamydia* spp. showed that proteins group into three distinct lineages, each strongly supported with posterior probabilities of 1.00, consisting of cluster 1: *C. abortus*, *C. felis*, *C. caviae* and *C. psittaci;* cluster 2: *C. pecorum* and *C. pneumoniae*; and cluster 3: *C. muridarum*, *C. trachomatis* and *C. suis* (Figure 4). Within each lineage, PmpD clustered by species with the exception of *C. suis* which clustered with *C. trachomatis* serovar E strains. Mapping of N-terminal peptides identified in *C. abortus* onto PmpD sequence alignments demonstrated the M-domain cleavage site ^1083^TLADIN to occur in a species-variable region located between two flanking regions of high sequence conservation (Figure S3a). The cleavage site identified by peptide EHNYSR^1445^ occurred in a region of high sequence conservation between all *Chlamydia* spp., whereas peptides LNCGMR^1518^ and ^215^LVFDGCE, located in the barrel- and passenger domains respectively, occurred in semi-conserved regions flanked by regions of high sequence conservation (Figure S3b and c). The cleavage site identified in the passenger domain by peptide ^67^EKPIHAQ occurred in a region of unique sequence composition in *C. abortus* (Figure S2d).

## Discussion

Although *pmp* gene carriage across *Chlamydia* spp. is variable through gene expansion and diversification in a number of *pmp* families, the genome of each species encodes only one *pmp*D gene. Our previous work has demonstrated that Pmp18D in *C. abortus* follows the same patterns of expression at both the transcript [25] and protein level [26] as PmpD of *C. trachomatis*. It was hypothesised that given these similarities in protein expression, the proteolytic processing of *C. abortus* Pmp18D would resemble that of the other previously studied PmpD molecules.

The results from previous studies on *C. trachomatis* [12], [27] and *C. pneumoniae* PmpD [28] together with those from this current study on *C. abortus* PmpD, demonstrate that there is a degree of heterogeneity in the processing of PmpD between species. At various time-points, full-length PmpD can be identified during the *C. trachomatis* developmental cycle. However, little if any full-length protein could be observed at any of the analysed time points in *C. abortus*, mirroring the observations made on the processing of the *C. pneumoniae* PmpD [28]. Due to differences in the timing of the chlamydial developmental cycle between species, direct comparison of specific time points is difficult and it cannot be discounted that full-length Pmp18D may have been observed with the analysis of additional time-points. However, the results from the current study suggest that virtually all of the Pmp18D in *C. abortus* is cleaved directly upon translocation to the outer surface of the bacterium, comprising the passenger domain with a molecular mass of approximately 104 kDa and a 48 kDa protein comprising the M-domain and the carboxy-terminal β-barrel domain. The majority of these products appear to remain associated with the outer membrane of the organism, as only a small proportion of the cleaved 104 kDa molecule could be identified in the soluble fraction.

Evidence from both Western blotting and proteomic methods has revealed further cleavage at the N-terminus of the 104 kDa protein. The Mid-Pmp18D antibody recognised an additional 3 protein bands, a single band of approximately 84 kDa and a doublet of 76 and 73 kDa that were not recognised by the N-terminal antibody. The smaller protein fragments cleaved from these proteins including the N-terminal 150aa polypeptide cleaved from the 104 kDa protein could not be subsequently identified in any cellular fraction. It has been previously hypothesised in *C. trachomatis* that some products of PmpD cleavage could act as soluble effector molecules [12]. These effectors could either impact on host transcription or cell lysis or be secreted from the host cell during the latter stages of the chlamydial developmental cycle. Certainly, the observed increase in the levels of the cleaved N-terminal proteins 72 h post-infection would fit with this hypothesis. However, while cleavage of the passenger domains appears to be a conserved feature of the PmpD molecule across *Chlamydia* spp., patterns of cleavage appear to be species-specific. Therefore, to understand whether the differences in cleavage patterns could be related to the presence of conserved regions or motifs within the molecule, an analysis of representative PmpD amino acid sequences was carried out to determine sequence similarity across species. Phylogenetic analysis of PmpD revealed a high degree of amino acid sequence heterogeneity between *Chlamydia* spp., particularly within the passenger domains. The identification of distinct clades of PmpD molecules comprising 3 clusters (Cluster 1: *C. abortus*, *C. psittaci*, *C. caviae* and *C. felis*; Cluster 2: *C. pneumoniae* and *C. pecorum* and Cluster 3: *C. trachomatis*, *C. suis* and *C. muridarum)*, could indicate a different function for PmpD for the species belonging to these different clusters. Indeed, in addition to difference in amino acid sequence and proteolytic processing, Swanson and colleagues [12] highlighted the presence of putative RGD and NLS domains within specific cleavage products of *C. trachomatis* PmpD [12]. These motifs are maintained in *C. suis* PmpD, which has 100% similarity in terms of amino acid sequence to that of *C. trachomatis* serovar E. However, neither of these motifs were identified in Pmp18D of *C. abortus*, and of the other sequenced members of the *Chlamydiaceae*, the NLS motif could only be identified in *C. felis*, although in a different location within the protein. The differences in cleavage patterns and the absence of conserved motifs in PmpD across species suggests that the protein may play different roles in different species of *Chlamydia*.

Cleavage of the PmpD molecule appears to be a conserved feature across the chlamydial species published to date. However, the patterns of proteolytic cleavage appear to be species-specific and phylogenetic analyses show large scale variation of PmpD within the *Chlamydiaceae*, with few conserved motifs between species in processing regions. Further studies are required to elucidate the function of PmpD and investigate whether this function is conserved across the different *Chlamydia* spp.

## Supporting Information

Figure S1 **Western blot demonstrating the solubility of the Pmp18D N-terminal passenger domain in 2% sarkosyl (using the Mid-Pmp18D antibody).** Lane 1 Whole *C. abortus* Elementary bodies (EBs); Lane 2 Soluble fraction after treatment of whole EBs with 2% sarkosyl; Lane 3 Soluble fraction after treatment of sarkosyl insoluble pellet with 1mM DTT/2% Sarkosyl; Lane 4 Sarkosyl insoluble material.
(PDF)

Figure S2 **Peptide coverage of Pmp18D protein cleavage products as determined by LC-ESI-MS-MS and MALDI ToF MS.**
(PDF)

Figure S3 **Region alignments of predicted cleavage sites in** ***C. abortus*** **Pmp18D.**
(DOC)
