# Supplementary figures and images for: Processing of Chlamydia abortus Polymorphic Membrane Protein 18D during the Chlamydial Developmental Cycle
Source: PLoS One. 2012 Nov 8;7(11):e49190. doi: 10.1371/journal.pone.0049190 (PMC3493501; doi:10.1371/journal.pone.0049190)

Figure S1

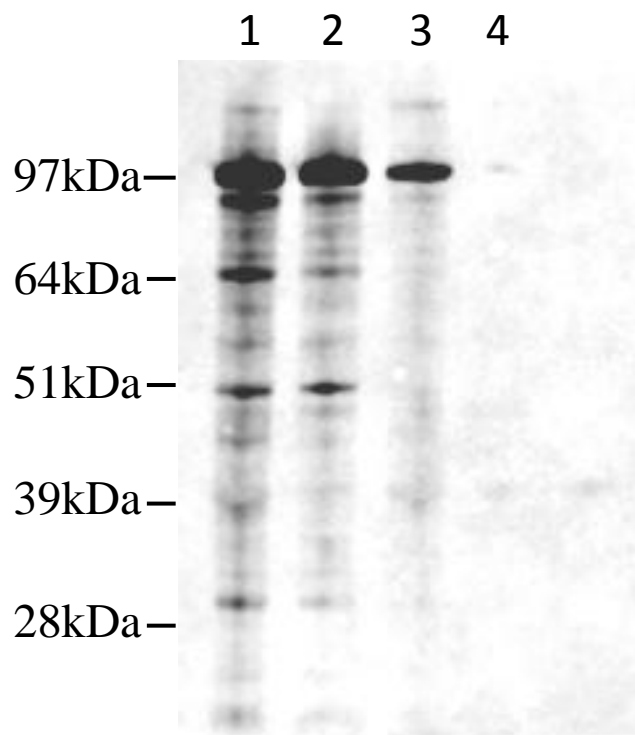

Supplement: Figure S1 — Western blot demonstrating the solubility of the Pmp18D N-terminal passenger domain in 2% sarkosyl (using the Mid-Pmp18D antibody). Lane 1 Whole C. abortus Elementary bodies (EBs); Lane 2 Soluble fraction after treatment of whole EBs with 2% sarkosyl; Lane 3 Soluble fraction after treatment of sarkosyl insoluble pellet with 1mM DTT/2% Sarkosyl; Lane 4 Sarkosyl insoluble material. (PDF) [file pone.0049190.s001.pdf]
