# Supplementary material for: Processing of Chlamydia abortus Polymorphic Membrane Protein 18D during the Chlamydial Developmental Cycle
Source: PLoS One. 2012 Nov 8;7(11):e49190. doi: 10.1371/journal.pone.0049190 (PMC3493501; doi:10.1371/journal.pone.0049190)
Supplement: Figure S2 — Peptide coverage of Pmp18D protein cleavage products as determined by LC-ESI-MS-MS and MALDI ToF MS. (PDF) [file pone.0049190.s002.pdf]

Figure S2

a)104kDa N-terminal passenger domain fragment

10	20	30	40	50	60
MIEKKVSRFQ	KSTFSHSVVL	AILVSTGMIT	NNDKLYGYVP	ASEVILDTLS	MPKAELEVPS
70	80	90	100	110	120
AGIFKK**EKPI**	**HAQGPK**KGET	DQETSLLDNT	STCVYK**VLVA**	**EDEQR**QHLEN	TSTIFQTCNV
130	140	150	160	170	180
LSWENLDTRS	TNAEAEKGKT	SPQYAVEDLQ	QGLAFCYKNA	PEHLLDANTP	GFLGIALKGT
190	200	210	220	230	240
RMK**SGLSFTN**	**LK**STAAGAAV	YSEEDVLFES	FKEK**LVFDGC**	**ESQAGGGAVS**	**GRSIAIHGCH**
250	260	270	280	290	300
**ALTIANSK**TD	VELKPTSGES	SDFSLGGGAF	NANQVHPVHK	SRFASGDVVF	LDNQGSVLLS
310	320	330	340	350	360
GNHADKANGG	AVACGNFICS	VNHSDIHYLD	NYALSGGAVS	SSKSMDFCGN	LGSIEFLNNQ
370	380	390	400	410	420
ALASSEGSLF	LGGGALAAGE	R**ISFLNNHGI**	**LCSK**NTAKCR	GGALLSREVR	IVENVGSSLF
430	440	450	460	470	480
KENTAEVTGG	AISSQHQVEI	DQNFGNVTFE	GNTSKFGGGA	IYCLLPAQPD	TDAQEPRIGS
490	500	510	520	530	540
GDIKIVDNVG	EVHFTSNANL	LDSQETHSYL	GGGALYGSNV	LISGNIGVIT	FSKNQAGQCE
550	560	570	580	590	600
SSSTHIGGGA	IFAHEVVTLS	GNSGEVTFSY	NKGQILPLPL	SPTPAEESST	SNAPIESSTP
610	620	630	640	650	660
VNLGVRGGGA	IFAKSISVED	NSAFVSFSEN	SMEIRDNQAQ	KENPLGGGAL	FGLDSVGLK**N**
670	680	690	700	710	720
**NVDLAFSNNR**	VSGGNSSGGA	ILSKEVAIAH	NGKVQFTRNC	AK**FLGGAVCA**	**LGDTLRIENN**
730	740	750	760	770	780
**ESTVSFVGNR**	TIAAGGALAS	AAGAVSISQN	LGKVEFKDNF	VFGDPYIENL	EKGQINSEGH
790	800	810	820	830	840
HSGGGAIFAK	**TSVVIRGNDN**	**K**VLFSGNSAG	CFGGAILTGS	LTSTESQERF	AAKVESDNTK
850	860	870	880	890	900
VVITENTGDV	IFSGNSTTAS	KHPEHNLFGG	GAIHTQDLII	K**NNEGSVAFY**	**NNYAPTGGAV**
910	920	930	940	950	960
**R**ISEK**GSVVL**	**QALGGDIVFQ**	**GNR**NSEDVSN	GMYFSGKESK	LVEVSAAGET	SVIFSDAIVF
970	980	990	1000	1010	1020
EDLTLRKGTK	DHEDALNDPT	LIFNSK**AKDD**	**AEITHSGNVR**	FSHATSKIPQ	VALLESGTLM
1030	1040	1050	1060	1070	1080
LSNK**AQLWLC**	**GLK**QEK**GSEI**	**LLSAGTVLRI**	**FDPNAKPTEK**	IESPTSNVYY	SAHESVKQPE
1090	1100	1110	1120	1130	1140
NKTLADINSI	GIDLASFVSS	DDETPVPPQI	IVPKGMTIGS	GSLDLNLLDS	VGAGYENHAL
1150	1160	1170	1180	1190	1200
LGKETDITLL	SFKSASSVLD	TPDLDHALEE	LRVKVSVPTI	TEDTYGHMGR	WSDPQVVDGK
1210	1220	1230	1240	1250	1260
LMINWKPTSY	KLNPEKSGAI	VLNSLWGHCG	DLRSLKQQQL	AHNITAQRME	LDFSTNIWGS
1270	1280	1290	1300	1310	1320
GMGTFTNCAT	IGKVDGFTHR	AGGYALGLDT	QLIEDFLIGG	SFAQFFGYTD	SQSYSSRSDQ
1330	1340	1350	1360	1370	1380
SGYLGSGYLG	IFTGSWLFKG	MLIYSNMHND	LNTQYPQPNL	GGSKGSWDSR	GILADAHVDY
1390	1400	1410	1420	1430	1440
RYIVNSRRLI	SSIVSAVVPF	VEAEYVYVNF	PKFTEIGSEA	RTFDEGHLQN	VAIPFGVTLE
1450	1460	1470	1480	1490	1500
HNYSRGQRSE	VNSVSFSYAI	DVYRQEPNVL	IHLPEASYSW	NGVGSNLARK	SMKAQFSNDT
1510	1520	1530	1540		
EWNSYFSTFL	GFTYEWREHT	IAYDLNCGMR	LIF		

b) 87.5kDa N-terminal passenger domain fragment

10	20	30	40	50	60
MIEKKVSRFQ	KSTFSHSVVL	AILVSTGMIT	NNDKLYGYVP	ASEVILDTLS	MPKAELEVPS
70	80	90	100	110	120
AGIFKKEKPI	HAQGPKKGET	DQETSLLDNT	STCVYKVLVA	EDEQRQHLEN	TSTIFQTCNV
130	140	150	160	170	180
LSWENLDTRS	TNAEAEKGKT	SPQYAVEDLQ	QGLAFCYKNA	PEHLLDANTP	GFLGIALKGT
190	200	210	220	230	240
RMKSGLSFTN	LKSTAAGAAV	YSEEDVLFES	FKEK**LVFDGC**	**ESQAGGGAVS**	**GRSIAIHGCH**
250	260	270	280	290	300
**ALTIANSK**TD	VELKPTSGES	SDFSLGGGAF	NANQVHPVHK	SRFASGDVVF	LDNQGSVLLS
310	320	330	340	350	360
GNHADKANGG	AVACGNFICS	VNHSDIHYLD	NYALSGGAVS	SSKSMDFCGN	LGSIEFLNNQ
370	380	390	400	410	420
ALASSEGSLF	LGGGALAAGE	R**ISFLNNHGI**	**LCSK**NTAKCR	GGALLSREVR	**IVENVGSSLF**
430	440	450	460	470	480
**K**ENTAEVTGG	AISSQHQVEI	DQNFGNVTFE	GNTSK**FGGGA**	**IYCLLPAQPD**	**TDAQEPR**IGS
490	500	510	520	530	540
GDIKIVDNVG	EVHFTSNANL	LDSQETHSYL	GGGALYGSNV	LISGNIGVIT	FSKNQAGQCE
550	560	570	580	590	600
SSSTHIGGGA	IFAHEVVTLS	GNSGEVTFSY	NKGQILPLPL	SPTPAEESST	SNAPIESSTP
610	620	630	640	650	660
VNLGVRGGGA	IFAKSISVED	NSAFVSFSEN	SMEIRDNQAQ	K**ENPLGGGAL**	**FGLDSVGLKN**
670	680	690	700	710	720
**NVDLAFSNNR**	VSGGNSSGGA	ILSK**EVAIAH**	**NGK**VQFTRNC	AK**FLGGAVCA**	**LGDTLRIENN**
730	740	750	760	770	780
**ESTVSFVGNR**	TIAAGGALAS	AAGAVSISQN	LGKVEFKDNF	VFGDPYIENL	EKGQINSEGH
790	800	810	820	830	840
HSGGGAIFAK	**TSVVIRGNDN**	**K**VLFSGNSAG	CFGGAILTGS	LTSTESQERF	AAKVESDNTK
850	860	870	880	890	900
VVITENTGDV	IFSGNSTTAS	KHPEHNLFGG	GAIHTQDLII	K**NNEGSVAFY**	**NNYAPTGGAV**
910	920	930	940	950	960
**R**ISEK**GSVVL**	**QALGGDIVFQ**	**GNR**NSEDVSN	GMYFSGKESK	LVEVSAAGET	SVIFSDAIVF
970	980	990	1000	1010	1020
EDLTLRKGTK	DHEDALNDPT	LIFNSK**AKDD**	**AEITHSGNVR**	FSHATSKIPQ	VALLESGTLM
1030	1040	1050	1060	1070	1080
LSNK**AQLWLC**	**GLK**QEK**GSEI**	**LLSAGTVLRI**	**FDPNAKPTEK**	IESPTSNVYY	SAHESVKQPE
1090	1100	1110	1120	1130	1140
NKTLADINSI	GIDLASFVSS	DDETPVPPQI	IVPKGMTIGS	GSLDLNLLDS	VGAGYENHAL
1150	1160	1170	1180	1190	1200
LGKETDITLL	SFKSASSVLD	TPDLDHALEE	LRVKVSVPTI	TEDTYGHMGR	WSDPQVVDGK
1210	1220	1230	1240	1250	1260
LMINWKPTSY	KLNPEKSGAI	VLNSLWGHCG	DLRSLKQQQL	AHNITAQRME	LDFSTNIWGS
1270	1280	1290	1300	1310	1320
GMGTFTNCAT	IGKVDGFTHR	AGGYALGLDT	QLIEDFLIGG	SFAQFFGYTD	SQSYSSRSDQ
1330	1340	1350	1360	1370	1380
SGYLGSGYLG	IFTGSWLFKG	MLIYSNMHND	LNTQYPQPNL	GGSKGSWDSR	GILADAHVDY
1390	1400	1410	1420	1430	1440
RYIVNSRRLI	SSIVSAVVPF	VEAEYVYVNF	PKFTEIGSEA	RTFDEGHLQN	VAIPFGVTLE
1450	1460	1470	1480	1490	1500
HNYSRGQRSE	VNSVSFSYAI	DVYRQEPNVL	IHLPEASYSW	NGVGSNLARK	SMKAQFSNDT
1510	1520	1530	1540		
EWNSYFSTFL	GFTYEWREHT	IAYDLNCGMR	LIF		

c) 48kDa carboxy terminal fragment

10	20	30	40	50	60
MIEKKVSRFQ	KSTFSHSVVL	AILVSTGMIT	NNDKLYGYVP	ASEVILDTLS	MPKAELEVPS
70	80	90	100	110	120
AGIFKKEKPI	HAQGPKKGET	DQETSLLDNT	STCVYKVLVA	EDEQRQHLEN	TSTIFQTCNV
130	140	150	160	170	180
LSWENLDTRS	TNAEAEKGKT	SPQYAVEDLQ	QGLAFCYKNA	PEHLLDANTP	GFLGIALKGT
190	200	210	220	230	240
RMKSGLSFTN	LKSTAAGAAV	YSEEDVLFES	FKEKLVFDGC	ESQAGGGAVS	GRSIAIHGCH
250	260	270	280	290	300
ALTIANSKTD	VELKPTSGES	SDFSLGGGAF	NANQVHPVHK	SRFASGDVVF	LDNQGSVLLS
310	320	330	340	350	360
GNHADKANGG	AVACGNFICS	VNHSDIHYLD	NYALSGGAVS	SSKSMDFCGN	LGSIEFLNNQ
370	380	390	400	410	420
ALASSEGSLF	LGGGALAAGE	RISFLNNHGI	LCSKNTAKCR	GGALLSREVR	IVENVGSSLF
430	440	450	460	470	480
KENTAEVTGG	AISSQHQVEI	DQNFGNVTFE	GNTSKFGGGA	IYCLLPAQPD	TDAQEPRIGS
490	500	510	520	530	540
GDIKIVDNVG	EVHFTSNANL	LDSQETHSYL	GGGALYGSNV	LISGNIGVIT	FSKNQAGQCE
550	560	570	580	590	600
SSSTHIGGGA	IFAHEVVTLS	GNSGEVTFSY	NKGQILPLPL	SPTPAEESST	SNAPIESSTP
610	620	630	640	650	660
VNLGVRGGGA	IFAKSISVED	NSAFVSFSEN	SMEIRDNQAQ	KENPLGGGAL	FGLDSVGLK**N**
670	680	690	700	710	720
**NVDLAFSNNR**	VSGGNSSGGA	ILSKEVAIAH	NGKVQFTRNC	AKFLGGAVCA	LGDTLRIENN
730	740	750	760	770	780
ESTVSFVGNR	TIAAGGALAS	AAGAVSISQN	LGKVEFKDNF	VFGDPYIENL	EKGQINSEGH
790	800	810	820	830	840
HSGGGAIFAK	TSVVIRGNDN	KVLFSGNSAG	CFGGAILTGS	LTSTESQERF	AAKVESDNTK
850	860	870	880	890	900
VVITENTGDV	IFSGNSTTAS	KHPEHNLFGG	GAIHTQDLII	KNNEGSVAFY	NNYAPTGGAV
910	920	930	940	950	960
RISEKGSVVL	QALGGDIVFQ	GNRNSEDVSN	GMYFSGKESK	LVEVSAAGET	SVIFSDAIVF
970	980	990	1000	1010	1020
EDLTLRKGTK	DHEDALNDPT	LIFNSKAKDD	AEITHSGNVR	FSHATSKIPQ	VALLESGTLM
1030	1040	1050	1060	1070	1080
LSNKAQLWLC	GLKQEKGSEI	LLSAGTVLRI	FDPNAKPTEK	IESPTSNVYY	SAHESVKQPE
1090	1100	1110	1120	1130	1140
NK**TLADINSI**	**GIDLASFVSS**	**DDETPVPPQI**	**IVPKGMTIGS**	**GSLDLNLLDS**	**VGAGYENHAL**
1150	1160	1170	1180	1190	1200
**LGKETDITLL**	**SFKSASSVLD**	**TPDLDHALEE**	**LR**VK**VSVPTI**	**TEDTYGHMGR**	**WSDPQVVDGK**
1210	1220	1230	1240	1250	1260
**LMINWKPTSY**	**K**LNPEK**SGAI**	**VLNSLWGHCG**	**DLR**SLK**QQQL**	**AHNITAQR**ME	LDFSTNIWGS
1270	1280	1290	1300	1310	1320
GMGTFTNCAT	IGKVDGFTHR	AGGYALGLDT	QLIEDFLIGG	SFAQFFGYTD	SQSYSSR**SDQ**
1330	1340	1350	1360	1370	1380
**SGYLGSGYLG**	**IFTGSWLFKG**	**MLIYSNMHND**	**LNTQYPQPNL**	**GGSK**GSWDSR	**GILADAHVDY**
1390	1400	1410	1420	1430	1440
**R**YIVNSRR**LI**	**SSIVSAVVPF**	**VEAEYVYVNF**	**PKFTEIGSEA**	**RTFDEGHLQN**	**VAIPFGVTLE**
1450	1460	1470	1480	1490	1500
**HNYSR**GQR**SE**	**VNSVSFSYAI**	**DVYRQEPNVL**	**IHLPEASYSW**	**NGVGSNLAR**K	SMKAQFSNDT
1510	1520	1530	1540		
EWNSYFSTFL	GFTYEWR**EHT**	**IAYDLNCGMR**	LIF		

d) 39.5kDa carboxy terminal fragment

10	20	30	40	50	60
MIEKKVSRFQ	KSTFSHSVVL	AILVSTGMIT	NNDKLYGYVP	ASEVILDTLS	MPKAELEVPS
70	80	90	100	110	120
AGIFKKEKPI	HAQGPKKGET	DQETSLLDNT	STCVYKVLVA	EDEQRQHLEN	TSTIFQTCNV
130	140	150	160	170	180
LSWENLDTRS	TNAEAEKGKT	SPQYAVEDLQ	QGLAFCYKNA	PEHLLDANTP	GFLGIALKGT
190	200	210	220	230	240
RMKSGLSFTN	LKSTAAGAAV	YSEEDVLFES	FKEKLVFDGC	ESQAGGGAVS	GRSIAIHGCH
250	260	270	280	290	300
ALTIANSKTD	VELKPTSGES	SDFSLGGGAF	NANQVHPVHK	SRFASGDVVF	LDNQGSVLLS
310	320	330	340	350	360
GNHADKANGG	AVACGNFICS	VNHSDIHYLD	NYALSGGAVS	SSKSMDFCGN	LGSIEFLNNQ
370	380	390	400	410	420
ALASSEGSLF	LGGGALAAGE	RISFLNNHGI	LCSKNTAKCR	GGALLSREVR	IVENVGSSLF
430	440	450	460	470	480
KENTAEVTGG	AISSQHQVEI	DQNFGNVTFE	GNTSKFGGGA	IYCLLPAQPD	TDAQEPRIGS
490	500	510	520	530	540
GDIKIVDNVG	EVHFTSNANL	LDSQETHSYL	GGGALYGSNV	LISGNIGVIT	FSKNQAGQCE
550	560	570	580	590	600
SSSTHIGGGA	IFAHEVVTLS	GNSGEVTFSY	NKGQILPLPL	SPTPAEESST	SNAPIESSTP
610	620	630	640	650	660
VNLGVRGGGA	IFAKSISVED	NSAFVSFSEN	SMEIRDNQAQ	KENPLGGGAL	FGLDSVGLKN
670	680	690	700	710	720
NVDLAFSNNR	VSGGNSSGGA	ILSKEVAIAH	NGKVQFTRNC	AKFLGGAVCA	LGDTLRIENN
730	740	750	760	770	780
ESTVSFVGNR	TIAAGGALAS	AAGAVSISQN	LGKVEFKDNF	VFGDPYIENL	EKGQINSEGH
790	800	810	820	830	840
HSGGGAIFAK	TSVVIRGNDN	KVLFSGNSAG	CFGGAILTGS	LTSTESQERF	AAKVESDNTK
850	860	870	880	890	900
VVITENTGDV	IFSGNSTTAS	KHPEHNLFGG	GAIHTQDLII	KNNEGSVAFY	NNYAPTGGAV
910	920	930	940	950	960
RISEKGSVVL	QALGGDIVFQ	GNRNSEDVSN	GMYFSGKESK	LVEVSAAGET	SVIFSDAIVF
970	980	990	1000	1010	1020
EDLTLRKGTK	DHEDALNDPT	LIFNSKAKDD	AEITHSGNVR	FSHATSKIPQ	VALLESGTLM
1030	1040	1050	1060	1070	1080
LSNKAQLWLC	GLKQEKGSEI	LLSAGTVLRI	FDPNAKPTEK	IESPTSNVYY	SAHESVKQPE
1090	1100	1110	1120	1130	1140
NK**TLADINSI**	**GIDLASFVSS**	**DDETPVPPQI**	**IVPKGMTIGS**	**GSLDLNLLDS**	**VGAGYENHAL**
1150	1160	1170	1180	1190	1200
**LGK**ETDITLL	SFKSASSVLD	TPDLDHALEE	LRVKVSVPTI	TEDTYGHMGR	WSDPQVVDGK
1210	1220	1230	1240	1250	1260
LMINWKPTSY	KLNPEK**SGAI**	**VLNSLWGHCG**	**DLR**SLK**QQQL**	**AHNITAQR**ME	LDFSTNIWGS
1270	1280	1290	1300	1310	1320
GMGTFTNCAT	IGKVDGFTHR	AGGYALGLDT	QLIEDFLIGG	SFAQFFGYTD	SQSYSSRSDQ
1330	1340	1350	1360	1370	1380
SGYLGSGYLG	IFTGSWLFKG	MLIYSNMHND	LNTQYPQPNL	GGSKGSWDSR	**GILADAHVDY**
1390	1400	1410	1420	1430	1440
**R**YIVNSRR**LI**	**SSIVSAVVPF**	**VEAEYVYVNF**	**PKFTEIGSEA**	**R**TFDEGHLQN	VAIPFGVTLE
1450	1460	1470	1480	1490	1500
HNYSRGQRSE	VNSVSFSYAI	DVYRQEPNVL	IHLPEASYSW	NGVGSNLARK	SMKAQFSNDT
1510	1520	1530	1540		
EWNSYFSTFL	GFTYEWREHT	IAYDLNCGMR	LIF		
