# Supplementary material for: Processing of Chlamydia abortus Polymorphic Membrane Protein 18D during the Chlamydial Developmental Cycle
Source: PLoS One. 2012 Nov 8;7(11):e49190. doi: 10.1371/journal.pone.0049190 (PMC3493501; doi:10.1371/journal.pone.0049190)
Supplement: Figure S3 — Region alignments of predicted cleavage sites in C. abortus Pmp18D. (DOC) [file pone.0049190.s003.doc]

**Figure S3**

**a) ^1083^TLADIN**

C. abortus S26/3 [CAH64218] GTLMLSNKAQ LWLCGLKQEK GSEILLSAGT VLRIFD.... .....PNAKP TEKIESPTSN VYYSAHESVK

C. abortus CPD13 [ACN69114] GTLMLSNKAQ LWLCGLKQEK GSEILLSAGT VLRIFD.... .....PNAKP TEKIESPTSN VYYSAHESVK

C. abortus CP16 [ACN69113] GTLMLSNKAQ LWLCGLKQEK GSEILLSAGT VLRIFD.... .....PNAKP TEKIKSPTSK VYYSAYESVK

C. abortus 1B [ABY41240] GTLMLSNKAQ LWLCGLKQEK GSEILLSAGT VLRIFD.... .....PNAKP TEKIESPTSN VYYSAHESVK

C. psittaci 6BC [ADZ18878] GTLVLSNKAQ LWLCGLKQER GSEILLSAGT VLRIFD.... .....PNAKP AEKIESPTSK VYYSAYDPVK

C. caviae GPIC [AAP05547] GTLILSDNAE LWLCGLKQEK GSEILLSAGT VLRIFD.... .....PNAKP EEKPESPSAR SYYSAYDSAR

C. felis Fe/C-56 [BAE80981] GTLVLSDKAQ LWLCGLKQEK GSEILLSVGT VLRIFD.... .....PNVKS VEKIESPSAK SYYSNYEIEK

C. pneumoniae AR39 [AAF38684] GTLALSQNAE LWLAGLKQET GSSIVLSAGS ILRIFDSQVD SSAPLPTENK EETLVSAGVQ INMSSPTPNK

C. pecorum E58 [AEB41103] GTLALTCQAQ LWLGGLKQEP KSDILMEAGT VLRVCE.... ..QFLKNEQK SEGREHPETP PFVEAAVLFP

C. suis [ACL83363] GSLELLNGAT LCSYGFKQDA GAKLVLASGS KLKILDSGTP V.QGHAVSKP EAEIESSSEP EGAHSLWIAK

C. trachomatis D-LC [ADI52498] GSLELLNGAT LCSYGFKQDA GAKLVLAAGA KLKILDSGTP VQQGHAISKP EAEIESSSEP EGAHSLWIAK

C. muridarum Nigg [AAF39070] GGLELLHGAI LCSYGVKQDP RAKIVLSAGS KLKILD.... ..SEQENNAE IGDLEDSVNS EKTPSLWIGK

C. abortus S26/3 [CAH64218] QPENK**T..LA D......IN**S IGIDLASFVS SDDE.TPVPP QIIVPKGMTI GSGSLDLNLL DSVGAGYENH

C. abortus CPD13 [ACN69114] QPENKT..LA D......INS IGIDLASFVS SDDE.TPVPP QIIVPKGMTI GSGSLDLNLL DSVGAGYENH

C. abortus CP16 [ACN69113] QPENKT..LA D......INS IGIDLASFVS SDDE.TPLPP QIIVPKGMTI GSGSLDLNLL DSAGAGYENH

C. abortus 1B [ABY41240] QPENKT..LA D......INS IGIDLASFVS SDDE.TPVPP QIIVPKGMTI GSGSLDLNLL DSVGAGYENH

C. psittaci 6BC [ADZ18878] NPGKKT..LA D......INS IGIDLASFVS SDDE.TPLPP QIIVPQGMTI GSGSLDLNLL DSAGAGYENH

C. caviae GPIC [AAP05547] NPEEKT..LA D......ISV IGVDLASFVA SEDEAAPLPP QIIVPKGTTI GSGSLDLNLV DSAGVGYENH

C. felis Fe/C-56 [BAE80981] NPIEKT..LA D......ISS IGVDLASFVT NDDGSSPLPP QIIVPKGTTI GSGSLDLSLV DSDGAGYENH

C. pneumoniae AR39 [AAF38684] DKAVDTPVLA D......IIS ITVDLSSFVP EQDGTLPLPP EIIIPKGTKL HSNAIDLKII DPTNVGYENH

C. pecorum E58 [AEB41103] KASVETTEAA SSSESIVLSN LSIDISSFAS EINS.EALPP KLILPEGSID QLKNLRLKVV DVEGLGYENH

C. suis [ACL83363] NAQTTVP.MV D......IHT ISVDLASFSS SQQEGTVEAP QVIVPGGSYV RSGELNLELV NTTGTGYENH

C. trachomatis D-LC [ADI52498] NAQTTVP.MV D......IHT ISVDLASFSS SQQEGTVEAP QVIVPGGSYV RSGELNLELV NTTGTGYENH

C. muridarum Nigg [AAF39070] NAQAKVP.LV D......IHT ISIDLASFSS KAQETPEEAP QVIVPKGSCV HSGELSLELV NTTGKGYENH

C. abortus S26/3 [CAH64218] ALLGKETDIT

C. abortus CPD13 [ACN69114] ALLGKETDIT

C. abortus CP16 [ACN69113] ALLGKETDIT

C. abortus 1B [ABY41240] ALLGKETDIT

C. psittaci 6BC [ADZ18878] ALLRKETDIT

C. caviae GPIC [AAP05547] ALLNKETDIT

C. felis Fe/C-56 [BAE80981] ALLNKETDLT

C. pneumoniae AR39 [AAF38684] ALLSSHKDIP

C pecorum E58 [AEB41103] SLLSHSRNIS

C. suis [ACL83363] ALLKNEAKVP

C. trachomatis D-LC [ADI52498] ALLKNEAKVP

C. muridarum Nigg [AAF39070] ALLKNDTQVS

**b) LNCGMR^1518^**

C. abortus S26/3 [CAH64218] VVPFVEAEYV YVNFPKFTEI GSEARTFDEG HLQNVAIPFG VTLEHNYSRG QRSEVNSVSF SYAIDVYRQE

C. abortus CPD13 [ACN69114] VVPFVEAEYV YVNFPKFTEI GSEARTFDEG HLQNVAIPFG VTLEHNYSRG QRSEVNSVSF SYAIDVYRQE

C. abortus CP16 [ACN69113] VVPFVEAEYV YVNFPKFTEI GSEARTFDEG HLQNIAIPFG VTLEHNYSLG QRSEVNSVSF SYAIDIYRQE

C. abortus 1B [ABY41240] VVPFVEAEYV YVNFPKFTEI GSEARTFDEG HLQNVAIPFG VTLEHNYSRG QRSEVNSVSF SYAIDVYRQE

C. psittaci 6BC [ADZ18878] VVPFVEAEYV YVDFPKFTEV GSEARTFGEG HLQNVAIPFG VTLEHNYSRG QRSEVNSFSF SYAIDVYRQE

C. caviae GPIC [AAP05547] VVPFVEAEYV YIDLPTFAEV GSEVRTFAEG HLQNIAIPFG ITLEHNYSRG QRSEVNSLSF SYALDVYRKA

C. felis Fe/C-56 [BAE80981] VVPFVEAEYT YMDLPAFTET GDEIRSFAEG HLQNVTIPFG LTLEHNYSRG QRSEVNSLSF SYALDVYRWE

C. pneumoniae AR39 [AAF38684] VVPFVEAEYV RIDLPEISEQ GKEVRTFQKT RFENVAIPFG FALEHAYSRG SRAEVNSVQL AYVFDVYRKG

C. pecorum E58 [AEB41103] YVPFVEVEYV RVDLPEVQEF GYEARTFQPS RLENVAIPFG ITLEQGYARG SRSEVNGFSV AYVLDVYRKQ

C. suis [ACL83363] FNPYVEVSYA SMKFPGFTEQ GREARSFEDA SLTNITIPLG MKFELAFIKG QFSEVNSLGI SYAWEAYRKV

C. trachomatis D-LC [ADI52498] FNPYVEVSYA SMKFPGFTEQ GREARSFEDA SLTNITIPLG MKFELAFIKG QFSEVNSLGI SYAWEAYRKV

C. muridarum Nigg [AAF39070] FNPYVEVSYA SAKFPSFVEQ GGEARAFEET SLTNITVPFG MKFELSFTKG QFSETNSLGI GCAWEMYRKV

C. abortus S26/3 [CAH64218] PNVLIHLPEA SYSWNGVGSN LARKSMKAQF SNDTEWNSYF STFLGFT--- --YEWREHTI AYD**LNCGMR**L IF

C. abortus CPD13 [ACN69114] PNVLIHLPEA SYSWNGVGSN LARKSMKAQF SNDTEWNSYF STFLGFT--- --YEWREHTI AYDLNCGMRL IF

C. abortus CP16 [ACN69113] STVLIHLPEA SYSWNGVGSS LSRKSMKAQF SNDTEWNSYF STFLGFT--- --YEWREHTI AYDLNCGMRL IF

C. abortus 1B [ABY41240] PNVLIHLPEA SYSWNGVGSN LARKSMKAQF SNDTEWNSYF STFLGFT--- --YEWREHTI AYDLNCGMRL IF

C. psittaci 6BC [ADZ18878] PTVLINLPEA SYSWDGVGSN LSRKSIKAQF SNDTEWNSYF STFLGFT--- --YEWREHTI AYDLNCGMRL IF

C. caviae GPIC [AAP05547] PTVLINLPAA SYSWEGVGSD LSRKFMKAQF SNDTEWSSYF STFLGFT--- --YEWREHTV SYDVNGGIRL IF

C. felis Fe/C-56 [BAE80981] PKVLINLPVA SYSWEGIGSD LARKSIKAQF SNDTEWNSYF STFLGAT--- --YEWREHTV SYDVNCGMRV IF

C. pneumoniae AR39 [AAF38684] PVSLITLKDA AYSWKSYGVD IPCKAWKARL SNNTEWNSYL STYLAFN--- --YEWREDLI AYDFNGGIRI IF

C. pecorum E58 [AEB41103] PETMISLPEA QFSWKGEGAD VFRKALRAQI NNDTEWCSYF STHFGVN--- --YEWREHMS TVDVNAGVRV IF

C. suis [ACL83363] EGGAVQLLEA GFDWEGAPMD LPRQELRVAL ENNTEWSSYF STVLGLTAFC GGFTSTASKL GYEANAGLRL IF

C. trachomatis D-LC [ADI52498] EGGAVQLLEA GFDWEGAPMD LPRQELRVAL ENNTEWSSYF STVLGLTAFC GGFTSTDSKL GYEANTGLRL IF

C. muridarum Nigg [AAF39070] EGRSVELLEA GFDWEGSPID LPKQELRVAL ENNTEWSSYF STALGVTAFC GGFSSMDNKL GYEANAGMRL IF

**c) ^215^LVFDGCE**

C. abortus S26/3 [CAH64218] RMKSGLSFTN LKSTAAGAAV YSEEDVLFES FKEK**LVFDGC E**SQAGGGAVS GRSIAIHGCH ALTIANSKTD

C. abortus CPD13 [ACN69114] RMKSGLSFTN LKSTAAGAAV YSEEDVLFES FKEKLVFDGC ESQAGGGAVS GRSIAIHGCH ALTIANSKTD

C. abortus CP16 [ACN69113] GMKSGLSLTN LKSTAAGAAV YSEEDVLFES FKEKLVFDGC ESQAGGGAVS GRSIAIHGCH AVTIANSKTD

C. abortus 1B [ABY41240] RMKSGLSFTN LKSTAAGAAV YSEEDVLFES FKEKLVFDGC ESQAGGGAVS GRSIAIHGCH ALTIANSKTD

C. psittaci 6BC [ADZ18878] GMKSGLSFTN LKSTAAGAAV YSDEDVVFES LKEKLVFDGC ESQAGGGAVS GRSIAIHGCH DVTIANSTTD

C. caviae GPIC [AAP05547] GSTSGLSFSN LKSLSAGSAV YSDEDVVFEH LKEKLFFEGC ESQAGGGAVS GRSIAINGCH DVSAVSCKTD

C. felis Fe/C-56 [BAE80981] GSKSGLSFSN LSSIASGAAV YSDEDVIFEH FKENLLFEGC ASQACGGAVS GRSIALNGCH DVSLLNCKSG

C. pneumoniae AR39 [AAF38684] GANSGLGFEN LKAPKSGAAV YSDRDIVFEN LVKGLSFISC ESLEDGSA.A GVNIVVTHCG DVTLTDCATG

C. pecorum E58 [AEB41103] EESSRMSFEN ISASKSGSAL YSDEDLIFKN LTGGVNLSNC ESLEDGG--- ---------- ----------

C. suis [ACL83363] SSKAGITLTD VKASLSGAAL YSTEDLIFEK IKGGLEFASC SSLEQGGACA AQSILIHDCQ GLQVKHCTTA

C. trachomatis D-LC [ADI52498] SSKAGITLTD VKASLSGAAL YSTEDLIFEK IKGGLEFASC SSLEQGGACA AQSILIHDCQ GLQVKHCTTA

C. muridarum Nigg [AAF39070] GSKDGITLTD IKSSLSGAAL YSSDDLIFER IKGDIELSSC SSLERGGACS AQSILIHDCQ GLTVKHCAAG

**d) ^67^EKPIHAQ and ^76^KGETDQ**

C. abortus S26/3 [CAH64218] VILDTLSMPK AELEVPSAGI F-------KK **EKPIHAQ**GPK **KGETDQ**ETSL LDNTSTCVYK VLVAE-DEQR

C. abortus CPD13 [ACN69114] VILDTLSMPK AELEVPSAGI F-------KK EKPIHAQGPK KGETDQETSL LDNTSTCVYK VLVAE-DEQR

C. abortus CP16 [ACN69113] VILDTLSMPK AELEVPSAGI F-------KK EKPIHAQEPK KGETDQETSL LDNTSTCVYK VLVAE-DEQR

C. abortus 1B [ABY41240] VILDTLSMPK AELEVPSAGI F-------KK EKPIHAQGPK KGETDQETSL LDNTSTCVYK VLVAE-DEQR

C. psittaci 6BC [ADZ18878] VILDTLSMPK AQLEAPSAGI F--------R KEKSHAQEPK KEETDVERSL LDNTSTCVYK VLVAE-DEQR

C. caviae GPIC [AAP05547] AFLSDSSL-K TQLETTSAGV F---RKVKST DTQEVQKENK EENTPVETSF IENASSCSVA ILGSE-CGQR

C. felis Fe/C-56 [BAE80981] VFLDTTSLSK PPVETTVAGI FGDKKKEKSS EKKNPKKENK EAETHVDTSL LHDISSCVTK ILGAD-VAQS

C. pneumoniae AR39 [AAF38684] VFNKQFEEHS AHVEEAQTSV L------KGS DPVNPSQKES EKVLYTQVPL TQGSSGESLD LADANFLEHF

C. pecorum E58 [AEB41103] VFADENTIDA ALL------- ---------- ---------- -----CSVEL LEGVSGKGLD LSS---LLQL

C. suis [ACL83363] ELVYVGPQAV LLLDQIRDLF V--------- -------GSK DSQAEGQYRL IVGDPSSFQE KD----ADTL

C. trachomatis D-LC [ADI52498] ELVYVGPQAV LLLDQIRDLF V--------- -------GSK DSQAEGQYRL IVGDPSSFQE KD----ADTL

C. muridarum Nigg [AAF39070] HPAYLIPQAG LLLDHIKDIF I--------- -------GPK DSQDKGQYKL IIGEAGSFQD SN----AETL
